# Precision (repeatability and reproducibility) of ocular parameters obtained by the Tomey OA-2000 biometer compared to the IOLMaster in healthy eyes

**DOI:** 10.1371/journal.pone.0193023

**Published:** 2018-02-27

**Authors:** Yanjun Hua, Wei Qiu, Qiuyi Xiao, Qiang Wu

**Affiliations:** Department of Ophthalmology, Shanghai Jiao Tong University Affiliated Sixth People’s Hospital, Xuhui District, Shanghai, China; Universidad de Monterrey Division de Ciencias de la Salud, MEXICO

## Abstract

**Purpose:**

To assess the precision (repeatability and reproducibility) of ocular parameters measured by the Tomey OA-2000 biometer, and to compare them with those measured by the IOLMaster.

**Methods:**

In this prospective study, the right eyes of 108 healthy subjects were included. Three consecutive scans were obtained by 2 observers using the Tomey OA-2000, and in the same session one observer used the IOLMaster (version 5.4.4.0006) for the measurements. About 1 week later, 3 scans were obtained by one observer using the Tomey OA-2000. The axial length (AL), central corneal thickness (CCT), anterior chamber depth (ACD), lens thickness (LT), keratometer readings, pupil diameter (PD) and corneal diameter (CD) values measured by the Tomey OA-2000 and IOLMaster were analyzed. The coefficient of variation (CoV), intraclass correlation coefficient (ICC), within subject standard deviation (Sw) and 2.77Sw were calculated to assess the repeatability and reproducibility. The paired t test and Bland-Altman plots were used to analyze the differences and agreements of parameters measured by the two devices, respectively.

**Results:**

Intraobserver repeatability, and interobserver and intersession reproducibility of the AL, CCT, ACD, LT, Kf, Ks, Km, PD and CD values measured by the Tomey OA-2000 biometer showed a CoV of less than 1% except that for PD, and an ICC of more than 0.97 except that for PD and CD. The AL, Kf, Ks, Km and CD values measured by the Tomey OA-2000 were 0.058 ± 0.094 mm, 0.088± 0.150 diopters (D), 0.163 ± 0.170 D, 0.127 ± 0.117 D and 0.171 ± 0.217 mm lower than those measured by the IOLMaster, respectively (all *Ps* < 0.05). However, the ACD values from the two devices were comparable (P = 0.169). The 95% linite of agreement (LoA) of the AL, ACD, CD and all keratometer readings were no more than 0.24 mm, 0.14 mm 0.60 mm and 0.5 D, respectively.

**Conclusion:**

Except for the PD and CD, the ocular parameters measured by the Tomey OA-2000 were highly repeatable and reproducible. Except for the CD value, there was good agreement of ocular parameters measured by the Tomey OA-2000 and the IOLMaster in healthy eyes.

## Introduction

Accurate ocular biometry is essential for predicting the intraocular lens (IOL) power before refractive and cataract surgery[1]. Generally for cataract surgery, precise ocular biometry can decrease refractive error and achieve the predicted postoperative outcome. Ultrasound-based axial length (AL) and anterior chamber depth (ACD, from corneal epithelium to anterior surface of crystalline lens) measurements have been the benchmark for a long period of time. However, the contact measurement is accompanied by the risk of infection and indentation compelling technicians and clinicians to seek safer and more comfortable techniques for ocular biometry. In 1999, the introduction of the partial coherence interferometry (PCI) based IOLMaster (Carl Zeiss Meditec AG) marked a great improvement due to its non-contact nature and higher resolution of AL measurement (about ±0.02 mm compared to ±0.15 mm by ultrasound)[2–4]. Furthermore, the inclusion of the corneal curvature, ACD and horizontal corneal diameter (CD) measurements in the same device, minimized the time for all measurements and improved the calculation of IOL power. In the last decade, the precision of the IOLMaster in both cataract biometry[5–7] and the study of refractive error evaluation[8, 9] set a new standard for ocular biometry. However, only AL measurement is based on PCI principle for the IOLMaster. The corneal curvature, ACD and CD are obtained from image analysis. Nevertheless, in IOLMaster 500 and older models, such as the model(software version: 5.4.4.0006) used in the present study, the corneal, crystalline lens or retinal thickness are not evaluated[10]. Additionally, realignment is required before each parameter is measured[11].

Several new devices based on optical low coherence reflectometry (OLCR)[12], such as Lenstar LS900 (Haag-Streit Köniz, Switzerland), Aladdin (Topcon, Japan), AL-Scan (Nidek, Japan) and OA-2000 (Tomey, Japan) are now commercially available for ocular biometry. In these devices, the AL, central corneal thickness (CCT), ACD (from corneal epithelium to anterior surface of crystalline lens), pupil diameter (PD) and crystalline lens thickness (LT) were measured automatically without the need of realignment. The Tomey OA-2000 biometer, which was an upgraded device of its predecessor OA-1000[13, 14], was developed to perform keratometry measurement based on Placido disc-based topography techniques with 9 rings each 256 points in a 5.5-mm zone projected on the anterior corneal surface[15]. Several studies[11, 16–24] have reported that the ocular parameters measured by Lenstar LS 900, Aladdin and AL-Scan biometers have excellent precision, and agreed well with those measured by the IOLMaster. To the best of our knowledge, few reports have been published to assess the precision (repeatability and reproducibility) of ocular parameters measured by Tomey OA-2000 biometer.

The purpose of this study was to evaluate the precision of the Tomey OA-2000 biometer for assessing ocular biometry, and to compare the parameters measured by the new device with those by IOLMaster.

## Subjects and methods

The study was conducted between July and September 2015 at the Department of Ophthalmology, Shanghai Jiao Tong University Affiliated Sixth People’s Hospital. The study protocol followed the principles of the Declaration of Helsinki and was approved by the Office of Research Ethics Committee, Shanghai Jiao Tong University Affiliated Sixth People’s Hospital. All subjects provided written informed consent after the purpose of the study had been explained to them in detail.

One-hundred and eight subjects (64 women), with a mean age of 27.43±7.37 years (range, 18–48 years) participated in this study. The mean spherical equivalent refraction was -3.24 ± 2.30 diopters (D; range, -8.88 to +2.50 D). All subjects could communicate well and cooperate with good fixation ability. The inclusion criteria were healthy subjects with a best corrected distance visual acuity equal to or better than 20/20 and an intraocular pressure of 10 to 21 mmHg. The exclusion criteria included 1) history of ocular pathology, or corneal or intraocular trauma; 2) previous ocular surgery; 3) wearing soft contact lenses within 2 weeks or rigid contact lenses within 4 weeks; 4) dry eye syndrome (with subjective dry eye symptoms, tear film break-up time shorter than 5 seconds). Only the right eye for each subject was selected for all measurements.

Repeatability, reproducibility and agreement were assessed based on standards adopted by the British Standards Institute and the International Organization for Standardization[25]. In the first session, 2 observers obtained 3 consecutive scans using the Tomey OA-2000 biometer, respectively, to assess intraobserver repeatability and interobserver reproducibility; ocular parameters were also measured by the IOLMaster (version 5.4.4.0006) in the same session. The average of AL, Keratometer readings, PD and CD obtained by the Tomey OA-2000 biometer by the first observer were used to compared with those obtained by IOLMaster. In the second session, about 1 week later, 1 observer obtained another 3 consecutive scans using the Tomey OA-2000 biometer to assess intersession reproducibility. All measurements were performed at least 3 hours after waking between 10 AM and 5 PM to minimize variations in the results. During the measurements, all the subjects sat in front of the devices, put their chin on the chinrest, focused on the target accordingly, opened their eyes wide after blinking before each scan. The whole procedure was completed within 15 minutes and only qualified measurements were adopted. All the subjects were confirmed to have avoided substantial reading before the measurements were performed.

### Instruments and measurements protocol

The Tomey OA-2000 biometer measures distances in the eye based on the OLCR principle using a superluminescent diode at a wavelength of 820 μm[15]. The AL, CCT, ACD, PD and LT can be measured using OLCR[22]. To measure the corneal curvature, Placido-disc based topography techniques with 9 rings each 256 points in a 5.5-mm zone are projected onto the cornea, and corneal curvature of 2-mm, 2.5-mm and 3-mm central corneal zone can be obtained[15]. The standard keratometric index of 1.3375 is used for calculating the keratometer readings[26, 27]. In the present study, the AL, CCT, ACD, PD, LT, CD and keratometer readings data of the 2.5 mm zone for each scan were collected.

The IOLMaster measures AL based on the PCI principle, which generates infrared light with a wavelength of 780 μm[10]. The AL is measured from the anterior corneal surface to retinal pigment epithelium. To measure the corneal curvature, the IOLMaster reflects 6 points of light from the tear film surface at a hexagonal pattern of approximately 2.3 mm diameter. The separation of opposite pairs of lights is measured by the internal software and the surface curvatures are calculated from 3 fixed meridians. The standard keratometric index of 1.3375 is used for calculating the Keratometer readings. To measure the ACD, the device directs a 0.7-mm wide slit of light beam through the anterior segment of the eye at an angle of 38 degrees to visual axis, and the distance between the anterior corneal pole and the anterior crystalline lens surface is calculated as the ACD[10]. In the present study, the AL, ACD, Keratometer readings and CD data were collected.

### Statistical analysis

Both SPSS software for Windows version 17 (SPSS Inc., Chicago, IL, U.S.) and MedCalc Statistical Software version 11.0 (MedCalc Software, Inc., Mariakerke, Belgium) were applied for statistical analyses. A P value of less than 0.05 was considered to have statistical significance. The distribution of all the datasets were analyzed for normality using Kolmogorov-Smirnov tests. No sample size calculation was made because statistical methods for agreement and precision studies recommend that the sample size should be at least 100 subjects[28]. To determine the intraobserver repeatability, interobserver and intersession reproducibility, within-subject standard deviation (Sw), test-retest repeatability (TRT), within-subject coefficient of variation (CoV), and intraclass correlation coefficient (ICC)[28] were calculated for the 3 consecutive measurements obtained during the first session. The test-retest repeatability was defined as 2.77Sw, which indicated the interval within which 95% of the differences between measurements are expected to lie. The CoV was calculated as the ratio of the Sw to the overall mean. A smaller CoV means that the repeatability was higher. The ICC (ranging from 0 to 1) assess the consistency for data sets of repeated measurements. The closer the ICC is to 1, the better the measurement consistency, and a value more than 0.9 indicates acceptable clinical reliability[29]. To compare ocular parameters obtained by the 2 devices, a paired t test was applied to identify pairs that had significant differences. Bland-Altman plots[25] were constructed to assess the agreement of measurements between the devices. The 95% limits of agreement (LoA) were defined as ±1.96 standard deviation. A narrower 95% LoA indicated better agreement between measurements.

## Results

### Intraobserver repeatability of ocular parameters measured by the Tomey OA-2000 biometer

Table 1 shows the repeatability of the AL, CCT, ACD, LT, Kf, Ks, Km, PD and CD values measured by the Tomey OA-2000 by the 2 observers. All CoV values were lower than 1.0%, except that of PD (5.298% and 4.889%). All ICC values were no less than 0.987, except that of PD (0.943 and 0.954) and CD (0.916 and 0.936). For both observers, the 2.77Sw for PD were 1.001 mm and 0.893 mm, while the 2.77Sw were 0.547 mm and 0.473 mm.

**Table 1:** Intraobserver repeatability of ocular parameters measured by Tomey OA-2000 biometer (n = 108).

Parameter	Observer	Mean ± SD	CoV (%)	Sw	2.77Sw	ICC
AL (mm)	1st	24.56 ± 1.30	0.034	0.013	0.037	1.000
	2nd	24.56 ± 1.30	0.035	0.011	0.030	1.000
CCT (μm)	1st	520.48 ± 31.63	0.668	5.08	14.06	0.991 (0.988–0.994)
	2nd	521.05 ± 31.69	0.591	4.75	13.17	0.993 (0.990–0.995)
ACD (mm)	1st	3.59 ± 0.32	0.426	0.020	0.057	0.999 (0.998–0.999)
	2nd	3.58 ± 0.32	0.385	0.016	0.045	0.999 (0.998–0.999)
LT (mm)	1st	3.72 ± 0.29	0.651	0.055	0.152	0.990 (0.987–0.993)
	2nd	3.73 ± 0.30	0.686	0.063	0.175	0.987 (0.982–0.991)
Kf (D)	1st	42.81 ± 1.44	0.141	0.070	0.200	0.999 (0.999–0.999)
	2nd	42.81 ± 1.41	0.140	0.077	0.214	0.999 (0.999–0.999)
Ks (D)	1st	43.84 ± 1.61	0.220	0.134	0.372	0.998 (0.997–0.998)
	2nd	43.84 ± 1.63	0.183	0.134	0.372	0.998 (0.997–0.998)
Km (D)	1st	43.32 ± 1.48	0.174	0.055	0.152	1.000 (0.999–1.000)
	2nd	43.32 ± 1.49	0.113	0.070	0.196	0.999 (0.999–1.000)
PD (mm)	1st	5.53 ± 0.88	5.298	0.363	1.001	0.943 (0.922–0.960)
	2nd	5.49 ± 0.87	4.885	0.322	0.893	0.954 (0.936–0.967)
CD (mm)	1st	11.88 ± 0.39	0.829	0.197	0.547	0.916 (0.885–0.940)
	2nd	11.87 ± 0.39	0.575	0.170	0.472	0.936 (0.912–0.954)

AL: axial length, CCT: central corneal thickness, ACD: anterior chamber depth, LT: lens thickness, Kf: flat keratometry, Ks: steep keratometry, Km: mean keratometry, PD: pupil diameter, CD: white to white, D: diopter, SD: standard deviation, CoV: coefficient of variance, Sw: within-subject standard deviation, ICC: intraclass correlation coefficient.

### Interobserver reproducibility of ocular parameters measured by the Tomey OA-2000 biometer

Table 2 shows the high interobserver reproducibility of the AL, CCT, ACD, LT, Kf, Ks, Km and CD values measured by the Tomey OA-2000, excluding PD. All CoV values were lower than 1% and all the ICC value were higher than 0.972, except that of PD (CoV = 3.855%, ICC = 0.944). The 2.77Sw values were 0.023 mm for AL, 8.355μm for CCT, 0.160 mm for ACD, 0.248 mm for CD and less than 0.210 D for all keratometer readings, while the 2.77Sw value for PD was 1.020 mm.

**Table 2:** Interobserver reproducibility of ocular parameters measured by Tomey OA-2000 biometer (n = 108).

Parameter	CoV (%)	Sw	2.77Sw	ICC
AL (mm)	0.022	0.008	0.023	1.000 (1.000–1.000)
CCT (μm)	0.423	3.016	8.355	0.995 (0.993–0.997)
ACD (mm)	0.332	0.022	0.061	0.998 (0.997–0.998)
LT (mm)	0.550	0.058	0.160	0.981 (0.972–0.987)
Kf (D)	0.082	0.045	0.124	0.999 (0.999–0.999)
Ks (D)	0.110	0.073	0.202	0.999 (0.999–0.999)
Km (D)	0.068	0.045	0.124	0.999 (0.999–0.999)
PD (mm)	3.855	0.370	1.020	0.944 (0.918–0.962)
CD (mm)	0.378	0.090	0.248	0.973 (0.961–0.982)

AL: axial length, CCT: central corneal thickness, ACD: anterior chamber depth, LT: lens thickness, Kf: flat keratometry, Ks: steep keratometry, Km: mean keratometry, PD: pupil diameter, CD: white to white, D: diopter, SD: standard deviation, CoV: coefficient of variance, Sw: within-subject standard deviation, ICC: intraclass correlation coefficient.

### Intrasession reproducibility of ocular parameters measured by Tomey OA-2000 biometer

Table 3 shows the high intrasession reproducibility of the AL, CCT, ACD, LT, Kf, Ks, Km and CD values measured by the Tomey OA-2000, excluding PD. All CoV values were lower than 1% and the ICC values were higher than 0.97, except that of PD (CoV = 4.787%, ICC = 0.903).

**Table 3:** Intrasession reproducibility of ocular parameters measured by Tomey OA-2000 biometer (n = 108).

Parameter	CoV (%)	Sw	2.77Sw	ICC
AL (mm)	0.061	0.072	0.199	0.999 (0.998–0.999)
CCT (μm)	0.466	3.213	8.900	0.995 (0.992–0.996)
ACD (mm)	0.374	0.023	0.065	0.997 (0.996–0.998)
LT (mm)	0.725	0.066	0.184	0.974 (0.962–0.982)
Kf (D)	0.082	0.050	0.139	0.999 (0.999–0.999)
Ks (D)	0.119	0.073	0.201	0.999 (0.999–0.999)
Km (D)	0.103	0.042	0.116	0.999 (0.999–0.999)
PD (mm)	4.787	0.365	1.010	0.903 (0.858–0.934)
CD (mm)	0.388	0.082	0.228	0.976 (0.964–0.983)

AL: axial length, CCT: central corneal thickness, ACD: anterior chamber depth, LT: lens thickness, Kf: flat keratometry, Ks: steep keratometry, Km: mean keratometry, PD: pupil diameter, CD: white to white, D: diopter, SD: standard deviation, CoV: coefficient of variance, Sw: within-subject standard deviation, ICC: intraclass correlation coefficient.

### Comparison of ocular parameters measured by the Tomey OA-2000 biometer and IOLMaster

**Fig 1:**
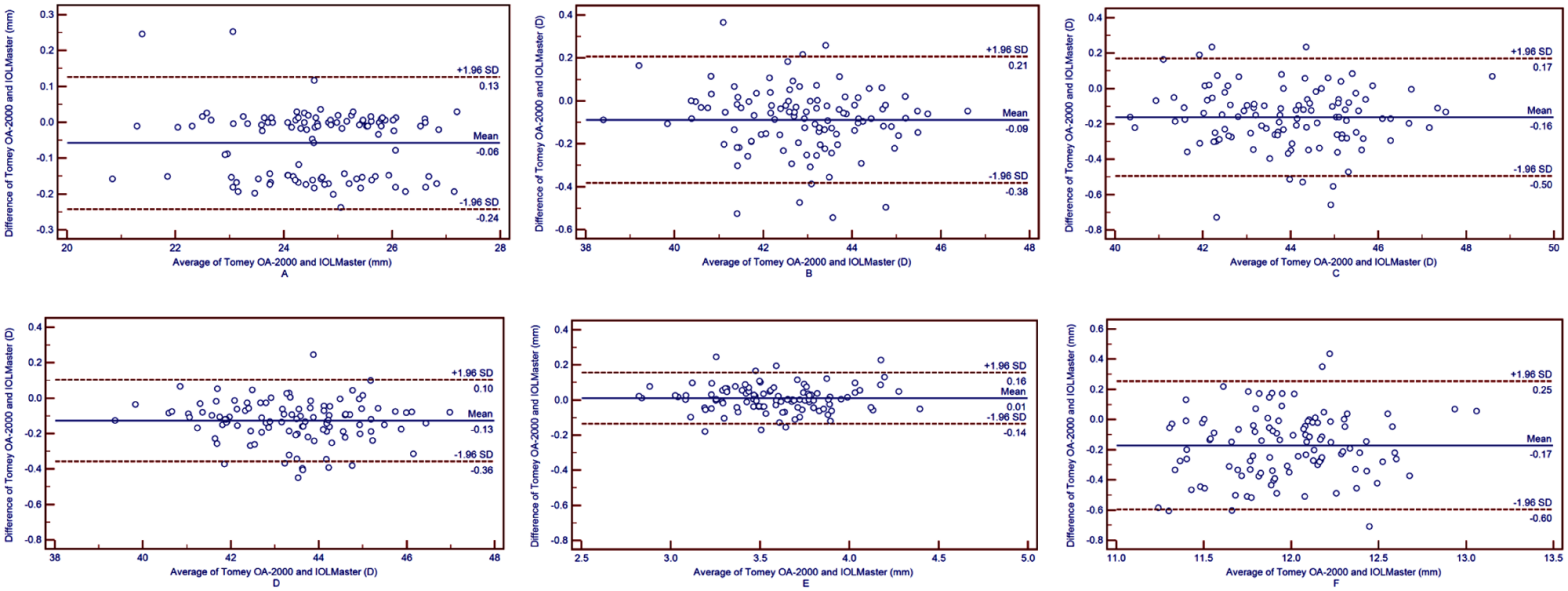
Bland-Altman plots present the mean plotted against the differences in values of AL (A), Kf (B), Ks (C), Km (D), ACD (E) and CD (F) for a comparison between the Toomey OA-2000 biometer and IOLMaster. The solid line indicates the mean difference. The interval between upper and lower lines represent the 95% LoA.

The average of the 3 measurements of AL, Keratometer readings, PD and CD by the Tomey OA-2000 were used to compared with those by IOLMaster. All ocular parameters measured by the Tomey OA-2000 were statistically smaller than those measured by the IOLMaster, excluding the ACD (Table 4). The AL and CD values measured by the Tomey OA-2000 were 0.058 ± 0.094 mm and 0.171 ± 0.217 mm smaller, respectively, than those measured by the IOLMaster, and the Kf, Ks and Km values measured by the Tomey OA-2000 were 0.088± 0.150 D, 0.163 ± 0.170 D and 0.127 ± 0.117 D lower, respectively, than those measured by the IOLMaster (all Ps < 0.05). However, the ACD values from the 2 devices were comparable (P = 0.169). The 95% LoA of the AL, ACD, CD and Keratometer readings were 0.24 mm, 0.14 mm, 0.60 mm and no more than 0.5 D, respectively (Fig 1). This indicates that agreements of the AL, ACD and all the keratometer readings measured by the two devices were relatively good.

**Table 4:** Comparison of ocular parameters measured by Tomey OA-2000 biometer and IOLMaster (n = 108).

Pairings	Mean Difference ± SD	95% CI	t Value	p* Value
AL	-0.058 ± 0.094	-0.076 to -0.040	-6.405	< 0.05
Kf	-0.088 ± 0.150	-0.117 to -0.060	-6.107	< 0.05
Ks	-0.163 ± 0.170	-0.195 to -0.130	-9.978	< 0.05
Km	-0.127 ± 0.117	-0.150 to -0.105	-11.281	< 0.05
ACD	0.010 ± 0.075	-0.004 to 0.024	1.382	0.169
CD	-0.171 ± 0.217	-0.213 to -0.130	-8.188	< 0.05

AL: axial length, CCT: central corneal thickness, ACD: anterior chamber depth, LT: lens thickness, Kf: flat keratometry, Ks: steep keratometry, Km: mean keratometry, PD: pupil diameter, CD: white to white, D: diopter, SD: standard deviation, CI: confidence interval,

*: two tailed.

## Discussion

With the development of phacoemulsification and IOL implantation in cataract and refractive lens surgery, accurate ocular biometry is essential for achieving the predicted postopervative refractive outcomes. In the present study, we evaluated the precision of ocular parameters measured by the Tomey OA-2000 biometer and found excellent intraobserver repeatability and interobserver and intersession reproducibility for all parameters except for PD and CD. Moreover, we compared the AL, keratometer readings, ACD and CD measured by the new device with those measured by the IOLMaster and found that the differences were very small but statistically significant.

To the best of our knowledge, there is few study to investigate the precision (repeatability and reproducibility) of ocular parameters measured by Tomey OA-2000 biometer in healthy eyes, comprehensively. However, several studies have assessed the precision of ocular biometers based on the OLCR principle. Goebels SC et al.[14] evaluated the reproducibility of the Tomey OA-1000 (the predecessor of the Tomey OA-2000) for measuring the AL, ACD and CCT, and the Cronbach’s α was 1.000 for AL, 0.979 for ACD and 0.999 for CCT. Shammas HJ et al.[16] evaluated the repeatability and reproducibility of the Lenstar LS 900, and reported that all ICCs were more than 0.9, except that for CD (0.849). In Huang J et al.’s study[23], for evaluating the repeatability and reproducibility of AL-Scan biometer, all the ICCs were more than 0.96 except for CD (0.834 and 0.843 for both observers), and all the CoV values were less than 0.5% except that for PD (5.46% and 4.79%, respectively) and CD (1.70% and 1.69%, respectively). Similar results were observed in Kola M et al.’s study[24] with all the ICCs of more than 0.93 except for PD (0.872 and 0.905) for the repeatability assessment. In Huang J et al.’s another study[21], the repeatability and reproducibility of parameters measured by Aladdin ocular biometer in healthy and cataract eyes were evaluated, all the ICCs were more than 0.94 except that for CD in cataract eyes (0.814 and 0.795, respectively).

In the present study, we compared the parameters measured by the new device with those measured by the IOLMaster. It was observed that AL, keratometer readings and CD measured by Tomey OA-2000 biometer were significantly lower than those measured by IOLMaster (all *Ps* < 0.05), except for ACD (*P* = 0.169). Goebels S et al.[15] measured 138 cataract eyes of 74 patients using Tomey OA-2000, Lenstar and IOLMaster. They found that AL with Tomey OA-2000 showed smaller mean values compared with IOLMaster and higher values compared with Lenstar (*P* < 0.001), and for ACD measurements Tomey OA-2000 showed higher mean values compared with Lenstar and IOLMaster (*P* < 0.001). In Goebels S et al.’s study, the corneal radii measured from the 3 devices were compared, and we could calculated keratometer readings accordingly. Following the formula: K = (1.3375–1.000)/corneal radii[30], The flat K measured by Tomey OA-2000, Lenstar and IOLMaster were 43.66 D, 43.27 D and 43.38 D; and the steep K measured by Tomey OA-2000, Lenstar and IOLMaster were 44.18 D, 44.41 D and 44.41 D. It indicated that Tomey OA-2000 showed the highest values for the flat K and lowest values for the steep K. This was different from the results from the present study in which for all the keratometer readings including Kf, Ks and Km Tomey OA-2000 showed lower values compared with IOLMaster. Besides, for ACD measurements Tomey OA-2000 was comparable with IOLMaster in the present study, while it showed higher values than IOLMaster in Goebels S et al.’s study. They might be attributed to the different subjects included in the studies. In the present study, we included normal subject with healthy eyes and the mean age of 27.43±7.37 years, while in Goebels S et al.’s study the patients, who were scheduled for cataract surgery, with the mean age of 71±12 years were enrolled.

However, in the present study, for AL measurements the difference between Tomey OA-2000 and IOLMaster was rather small (on average -0.058 ± 0.094 mm, 95%LoA -0.24 to 0.13 mm) but statistically significant. Several studies referred to the comparison of OLCR and PCI biometers have had similar results. In Holzer et al.’s study[18], AL measurements by Lenstar were highly correlated to those by IOLMaster. In Liampa et al.’s study[31], similar AL values were measured by Lenstar and IOLMaster. Hoffer et al.[32] found that for AL measurements IOLMaster showed small but significantly difference compared with Lenstar.

For corneal curvature measurement, Tomey OA-2000 biometer possesses Placido-disc based topography with 9 rings in a 5.5 mm zone, and the corneal curvature of 2 mm, 2.5 mm and 3 mm central corneal zone can be measured. In the present study, we collected the data from 2.5 mm zone, and we found that all the keratometer readings (including Kf, Ks and Km) measured by Tomey OA-2000 were significantly lower than those measured by IOLMaster. The differences were 0.09 D for Kf, 0.16 D for Ks and 0.12 D for Km, respectively. As we know that a difference of 1.0 D in keratometer readings leads to a difference of approximately 1.4 D in IOL power prediction. So, the difference of about 0.1 D in K value would result in a difference of 0.14 D in IOL power prediction, which is clinically negligible.

In the present study, the ACD measured by Tomey OA-2000 and IOLMaster were comparable with the 95% LoA of -0.14 mm to 0.16 mm. This was different from previous studies. In Goebels S et al.’s studies[13, 15], the differences of ACD measurements between Tomey OA-1000 and IOLMaster, Tomey OA-2000 and IOLMaster were 0.4 mm and 0.2 mm, respectively. Liampa et al.[31] and Holzer et al.[18] reported 0.2 mm and 0.16 mm differences of ACD measurements between Lenstar LS 900 and IOLMaster. In Hoffer KJ et al’s study[33], the Aladdin biometer obtained 0.16 mm (U.S) and 0.05 mm (China) deeper ACD than IOLMaster 500, and these differences were statistically significant. In Hoffer KJ et al’s another study[34], IOLMaster 700 obtained 0.03 mm deeper ACD than Lenstar LS 900. In Huang J et al’s latest study[35], Tomey OA-2000 was used to measure 65 right eyes of 65 healthy people, and the results showed that the repeatability and reproducibility of Tomey OA-2000 were excellent for AL, keratometer readings, ACD, LT and CD values and good agreement with IOLMaster for most parameters. And these findings were similar to the present study.

There are some limitations for this study. First, we only included normal subjects with healthy eyes for the precision and agreement assessment, and the results might be different in eyes with cataract. Second, we only compared the new device with IOLMaster for AL, keratometer readings, ACD and CD measurements, but the comparisons of parameters measured by the new device and the ultrasonic equipment were not mentioned. Third, we did not compare the predicted outcomes of IOL powers by the new device with those by IOLMaster, and this would be our further work in the future. Forth, the model of the IOLMaster used in the present study was relatively old, and different results maybe generated for the new models of IOLMaster (500 and 700).

In conclusion, the ocular parameters measured by Tomey OA-2000 biometer were highly repeatable and reproducible, except for PD and CD. Good agreements of ocular parameters(except for CD values) measured by Tomey OA-2000 biometer and IOLMaster were found in healthy eyes.

## Supporting information

S1 Data Precision of the Tomey OA-2000 biometer. (XLSX)

S2 Data Comparison between the Tomey OA-2000 and the IOLMaster. (XLSX)
